# Genomic diversity of Pseudomonas aeruginosa causing bacteremic pneumonia in an intensive care unit, with emergence of an OXA-796-producing NDM-1 ST773 isolate

**DOI:** 10.1099/mgen.0.001815

**Published:** 2026-08-06

**Authors:** Kihyun Lee, Hyemin Chung, Kuenyoul Park, Sang-Ho Choi, Heungsup Sung

**Affiliations:** 1 CJ Bioscience, Seoul, Republic of Korea; 2 Division of Infectious Diseases, Department of Internal Medicine, Chung-Ang University Gwangmyeong Hospital, Gwangmyeong, Republic of Korea; 3 Department of Laboratory Medicine, Hanyang University Seoul Hospital, Hanyang University College of Medicine, Seoul, Republic of Korea; 4 Department of Infectious Diseases, Asan Medical Center, University of Ulsan College of Medicine, Seoul, Republic of Korea; 5 Department of Laboratory Medicine, Asan Medical Center, University of Ulsan College of Medicine, Seoul, Republic of Korea

**Keywords:** bacteremic pneumonia, integron, NDM-1, OXA-796, *Pseudomonas aeruginosa*, ST773

## Abstract

*Pseudomonas aeruginosa* bacteremic pneumonia carries exceptionally high mortality, yet there is a paucity of genomic characterization of the strains causing this infection. We performed hybrid sequencing (Illumina and Oxford Nanopore) of 12 non-redundant *P. aeruginosa* isolates from patients with severe bacteremic pneumonia admitted to the intensive care unit of a tertiary hospital between 2015 and 2023. The 12 isolates were assigned to nine distinct sequence types, suggesting that severe bacteremic pneumonia can arise from diverse *P. aeruginosa* lineages rather than being dominated by a single specialized or high-risk clone. In the combined dataset of our isolates and publicly available Korean *P. aeruginosa* genomes, type III secretion system exotoxin genotypes *exoU* and *exoS* showed a mutually exclusive and phylogenetically segregated distribution, as previously reported, with both genotypes represented among bacteremic pneumonia isolates. Carbapenemase genes were detected in only one isolate, PA22 (ST773), which harboured *bla*_NDM-1_ together with *bla*_OXA-796_ and was the only isolate displaying phenotypic carbapenem resistance and multidrug resistance. To assess the clonal relationship between *bla*_NDM-1_-positive PA22 and the carbapenemase-negative ST773 isolate PA20 and to track the evolution of PA22 resistome within a broader epidemiological context, we investigated the population structure of a global ST773 dataset. Core genome MLST-based minimum spanning trees revealed a deep bifurcation within ST773, separating *bla*_NDM-1_-positive and carbapenemase-negative lineages. Korean ST773 isolates formed two distinct clusters within the NDM-1-positive lineage, with the PA22-containing cluster phylogenetically proximal to isolates from the United States. Within the NDM-1-positive Korean cluster, *bla*_OXA-796_ was located in conserved class 1 integron gene cassette arrays that exhibit ongoing structural diversification among closely related isolates, evidenced by variable integration of IS110 elements. Our findings demonstrate that severe bacteremic pneumonia arises from phylogenetically diverse *P. aeruginosa* lineages and provide genomic context for the NDM-1-producing ST773 clone that is rapidly emerging in Korea.

## Data Summary

The genome sequences generated in this study have been deposited in NCBI GenBank under BioProject accession number PRJNA1435884. Assembled genome sequences of *Pseudomonas aeruginosa* (*n*=46,994; accessed 27 December 2025) and associated metadata were obtained from the NCBI Pathogen Detection database and BioSample records, respectively (Table S1, available in the online Supplementary Material).

**Impact Statement**

*Pseudomonas aeruginosa* bacteremic pneumonia is among the most lethal forms of hospital-acquired infection. However, genomic studies of this pathogen have largely been restricted to carbapenem-resistant isolates, potentially obscuring the full spectrum of strains responsible for this severe disease. Here, we apply long-read sequencing to characterize *P. aeruginosa* isolates from severe bacteremic pneumonia regardless of carbapenem susceptibility, revealing that these infections are caused by phylogenetically diverse lineages with heterogeneous virulence and resistance profiles rather than by a single dominant clone. This finding demonstrates that genomic surveillance focused exclusively on carbapenem-resistant isolates may provide an incomplete picture of the strain-level epidemiology in severe invasive infections. Furthermore, by placing the NDM-1-producing ST773 isolate identified in this study within a global population structure of ST773 genomes, we delineate the lineage-level separation between NDM-1-positive and carbapenemase-negative ST773 populations and document ongoing integron-mediated resistome diversification within the NDM-1-positive ST773. Given that NDM-1-producing ST773 has rapidly emerged as the most prevalent carbapenemase-producing *P. aeruginosa* genotype in Korea, our detailed genomic characterization of this lineage illustrates the utility of long-read sequencing for surveillance and infection control of this high-risk clone.

## Introduction

Bacteremic pneumonia, defined as pneumonia accompanied by concurrent bloodstream infection, is associated with high mortality, particularly when caused by *Pseudomonas aeruginosa*. In a large cohort study, the in-hospital mortality of bacteremic pneumonia was 37.1%, and *P. aeruginosa* accounted for 10.6% of the cases and was identified as an independent predictor of mortality [1]. *P. aeruginosa* is a major gram-negative pathogen in hospital-acquired and ventilator-associated pneumonia (HAP/VAP), accounting for 7–20% of nosocomial pneumonia across surveillance studies [2–4]. Bacteremia occurs in 4–17% of *P. aeruginosa* HAP/VAP cases [5] and represents a severe complication, with mortality exceeding 50% [6, 7] and higher than that of *P. aeruginosa* bacteremia originating from sources other than pneumonia [7]. The high mortality associated with *P. aeruginosa* bacteremic pneumonia is partly linked to inadequate empirical antibiotic therapy, and appropriate combination therapy was shown to reduce mortality [6]. Therefore, there is a critical need for characterizing epidemiological trends of the *P. aeruginosa* strains causing this severe infection, particularly regarding their virulence and antimicrobial resistance (AMR) profiles.

The type III secretion system (T3SS) plays a central role in the dissemination of *P. aeruginosa* from the lungs to the bloodstream. In a murine pneumonia model, T3SS exotoxin secretion was a major determinant of lung injury, and strains secreting ExoU showed a significantly increased risk of bacteremia [8]. Clinically, the *exoU* genotype was an independent predictor of early mortality in a *P. aeruginosa* bacteremia cohort, yet was less prevalent among multidrug-resistant strains, suggesting an inverse relationship between virulence and resistance [9]. Most *P. aeruginosa* strains carry either *exoU* or *exoS* but rarely both or neither [10], and ExoU secretion is generally considered to have the greatest impact on virulence, while ExoS exerts an intermediate effect [11]. Notably, ExoS has also been shown to directly damage pulmonary epithelial cells, leading to localized disruption of the pulmonary-vascular barrier and facilitating bacterial dissemination into the bloodstream [12]. A recent large-scale whole-genome sequencing (WGS) study of 773 patients with *P. aeruginosa* bloodstream infections further demonstrated that biofilm-related genes and epidemic clones ST175 and ST235 were associated with mortality, while T3SS was associated with septic shock, confirming the prognostic potential of genomic virulence markers [13].

Hospital-acquired *P. aeruginosa* infections are challenging to treat due to frequent multidrug resistance. Concerns around AMR are rising with the observation of internationally disseminated high-risk clones that harbour transferable resistance genes, including extended-spectrum β-lactamases and carbapenemases [14]. In Korea, molecular epidemiological studies of *P. aeruginosa* have primarily focused on carbapenem-resistant *P. aeruginosa* (CRPA). The molecular epidemiology of CRPA in Korea has undergone a notable shift in recent years. In 2012, the *bla*_IMP-6_-harbouring ST235 clone was reported as the predominant genotype among carbapenemase-producing *P. aeruginosa* [15]. However, a nationwide surveillance study published in 2023 revealed a marked emergence of the *bla*_NDM-1_-harbouring ST773 clone [16]. A multi-centre study published in 2025 reported that only 17.5% of the CRPA isolates were susceptible to ceftazidime/avibactam, and none of the carbapenemase-producing isolates in ST773, ST644 or ST235 were susceptible, highlighting the extremely limited therapeutic options for CRPA in Korea [17].

However, these existing studies have been confined to carbapenem-resistant isolates and do not encompass the full genomic characteristics of *P. aeruginosa* strains causing severe clinical infections, particularly bacteremic pneumonia. Moreover, given the potential inverse relationship between virulence and AMR [18], analyses restricted to CRPA may not capture the comprehensive genomic landscape of strains responsible for severe infections. In this study, we first performed WGS on *P. aeruginosa* clinical isolates that caused concurrent severe pneumonia and bacteremia in the intensive care unit (ICU), aiming to characterize their molecular epidemiology and genetic profiles. Following the identification of a carbapenem-resistant and multidrug-resistant ST773 isolate within this resistance-unselected collection dominated by carbapenem-susceptible isolates, we further investigated the molecular epidemiological context of this isolate by examining its β-lactamase repertoire, mobile genetic element architecture and clonal relationship within the global ST773 population.

## Methods

### Study isolates

This study was based on a prospective cohort of ICU-admitted adults (≥16 years of age) with severe *P. aeruginosa* pneumonia with concurrent bacteremia, enrolled since March 2010 at a 2,700-bed tertiary referral centre with a 28-bed medical ICU in Seoul, South Korea. *P. aeruginosa* pneumonia was defined as pneumonia with *P. aeruginosa* isolated from respiratory specimens or blood cultures, and severe pneumonia was defined as pneumonia complicated by septic shock or requiring mechanical ventilation at the time of ICU admission [19]. From this cohort, all available *P. aeruginosa* isolates collected between April 2015 and August 2025 were included, with isolates that could not be recovered upon subculture or were no longer available in storage excluded; all included isolates were non-redundant, with one isolate per patient. This prospective cohort was approved by the Institutional Review Board of Asan Medical Center (IRB no. 2010–0079), with informed consent waived due to the observational nature of the study.

### Short-read and long-read sequencing

Genomic DNA was extracted from pure bacterial cultures using the Zymo Quick-DNA HMW MagBead Kit (Zymo Research). DNA concentration and quality were assessed using the Invitrogen Qubit dsDNA HS Assay Kit (Thermo Fisher Scientific) and the Agilent TapeStation 4200 and Femto Pulse system (Agilent Technologies), respectively. WGS libraries for Illumina and Oxford Nanopore sequencing were prepared using the NEBNext Ultra II FS DNA Library Prep Kit (New England Biolabs) and the Native Barcoding Kit 24 V14 (Oxford Nanopore Technologies), respectively. Illumina sequencing was performed on a NovaSeq 6000 platform using the S1 Reagent Kit v1.2 for 300 cycles, generating 150 bp paired-end reads. Nanopore sequencing was performed on a GridION platform using R10.4.1 flow cells with the SUP basecalling model.

### Genome assembly

Illumina sequencing reads were quality filtered and trimmed using Fastp v0.22.0 [20], requiring a minimum Phred quality score of 30 and a minimum read length of 100 bp. Nanopore reads were filtered using Nanoq v0.10.0 [21], requiring a minimum read quality score of 16 and a minimum read length of 1600 bp. Long-read assemblies were first generated using Flye v2.9.1 [22] with the ‘--nano-hq’ option and otherwise default parameters. When Flye produced circularized contigs for all replicons, assemblies were polished using Illumina reads with Polypolish v0.6.0 [23] and subsequently oriented using DNAapler v1.2.0 [24]. For isolates in which *Flye* did not resolve complete circular assemblies, we tried alternative assemblies using Autocycler v0.2.1 [25] and Hybracter v0.11.0 [26]. The assembly with higher contiguity (e.g. smaller number of contigs) was selected as the final assembly. Assemblies generated using Autocycler were additionally polished with Polypolish v0.6.0 and oriented with DNAapler v1.2.0.

### Collection of publicly available genome sequences

We collected all assembled genome sequences annotated as *P. aeruginosa* in the NCBI Pathogen Detection database (https://www.ncbi.nlm.nih.gov/pathogens; accessed 27 December 2025; *n*=46,994). We did not apply additional quality-based filtering because genome assemblies released on the Pathogen Detection database have already passed database-level quality controls, including screening for anomalous genome size, completeness based on wgMLST locus coverage, assembly contiguity (i.e. N50 >10,000 bp), contamination assessed by the Foreign Contamination Screen tool [27] and species identity check based on average nucleotide identity. The associated metadata were obtained from the Pathogen Detection Isolate Browser, and collection date and geographic location fields were manually curated for the ST773 genomes to ensure consistent formatting. The resulting metadata table for the species-wide genome dataset (*n*=46,994) is provided in Table S1. These additional genome sequences were annotated and typed using the same versions of software and databases described below for the analyses of our bacteremic pneumonia isolates. The species-wide dataset was used to calculate the overall prevalence of resistance and virulence genotypes, whereas the ST773 subset was utilized in detailed analyses of population structure, carbapenemase and *bla*OXA allele distributions and class 1 integron structures.

### Strain typing and phylogenetic analyses

MLST was assigned to each genome using MLST v2.23.0 (https://github.com/tseemann/mlst) with the *P. aeruginosa* scheme and database hosted by PubMLST (accessed 19 October 2025). For ST773 genomes, we performed cgMLST analysis using chewBBACA v3.5.3 [28]. We defined the cgMLST loci that were conserved in all (100%) ST773 genomes using the functions CreateSchema and ExtractCgMLST; then called alleles using the function AlleleCall. Minimum spanning trees were constructed from cgMLST allele profiles using the goeBURST full minimum spanning tree algorithm implemented in PHYLOViZ v2.0 [29] and visualized using Cytoscape v3.10.4 [30] with manually curated isolate metadata for node colouring.

For phylogenetic analysis of a Korean *P. aeruginosa* dataset comprising multiple sequence types, we used the genome sequence of *Pseudomonas paraeruginosa* ATCC 9027 (GCA_002601815.1) as an outgroup and *P. aeruginosa* DSM 50071 (GCA_001045685.1) as an in-group reference; both genomes were removed from the final tree during visualization. Public genomes in the species-wide dataset for which the geographic location was recorded as ‘South Korea’ (*n*=41) were combined with the 12 genomes from bacteremic pneumonia isolates collected in this study and subjected to phylogenetic analysis. To collect core gene sequences, we searched the representative allele sequences from a published *P. aeruginosa* cgMLST scheme [31] against the genome assemblies of Korean isolates using blastn v2.17.0 with the minimum query coverage and identity cutoffs of 80 and 50%, respectively. For each locus, we extracted the hit allele sequences when alignments passing the thresholds were present as single copy. Multiple sequence alignments for each locus were generated using MAFFT v7.505 [32] and concatenated into a single super-matrix. Maximum-likelihood phylogenetic tree was inferred using IQ-TREE v2.0.7 [33], using a transversion model with empirical base frequencies and a free rate model with 10 categories, as selected by ModelFinder. The resulting tree was rooted, sorted and trimmed using the ape package and visualized using ggtree.

### Resistome, virulome and mobile genetic element analysis

Gene prediction was performed using Prokka v1.14.6 [34]. AMR determinants were identified using NCBI AMRFinderPlus v4.0.23 [35] with the database version released on 16 July 2025, applying the organism-specific parameter for *P. aeruginosa* (-O Pseudomonas_aeruginosa). Virulence-associated genes were identified by blastp searches against the Virulence Factor Database [36] accessed on 16 January 2026. Sequence similarity searches were performed using DIAMOND v2.1.21 [37], applying thresholds of 90% amino acid identity and 80% coverage of the reference sequence. Functional annotation was further supplemented using eggNOG-mapper v2.0.1 [38] with the eggNOG database (accessed 19 March 2019) [39].

Plasmid replicon types were predicted from assembled contig sequences using the mob_recon module of MOB-suite v3.1.9 [40]. IntegronFinder v2.0.5 [41] was used to identify integron structures from the contig sequences. ISEScan 1.7.3 [42] was used to identify the insertion sequence (IS) elements from the contig sequences. To identify integrative and conjugative elements (ICEs) and composite transposons, reference sequences from ICEberg (January 2026 release) [43] and TnCentral (January 2026 release) [44] were aligned against the assembled genome sequences using blastn + v2.17.0.

## Results

### Genome sequencing and assembly

In this study, we analysed the genome sequences of 12 non-redundant *P. aeruginosa* isolates recovered from patients with severe bacteremic pneumonia between April 2015 and August 2025. Antimicrobial susceptibility tests on these 12 isolates indicated that one isolate (PA22) was resistant to nine out of 10 antibiotics tested, including imipenem and meropenem, while the other 11 isolates were susceptible to all tested antibiotics (Table S2). Long-read assembly using nanopore sequencing reads resulted in fully circularized genome assemblies consisting of a single chromosome and a single or no plasmid for seven isolates. For the other five isolates, assemblies with the smallest number of contigs were selected among three assembly workflows, resulting in 2–12 unclosed contigs. The genome sizes determined by these assemblies ranged from 6.3 to 7.2 Mb and four copies of the ribosomal RNA operons [45, 46] were recovered consistently across all assemblies (Table 1).

**Table 1.** Clinical isolates from *P. aeruginosa* bacteremic pneumonia and summary statistics of their genome sequencing and assembly

Isolate	Collection date (yyyy-mm)	Sequencing depth (Mbp)		Longest read length (Kbp)	Median read length (Kbp)	Assembly workflow used^*^	No. of contigs			Assembly size (bp)	No. of rRNA operons
		Illumina	Nanopore				Total	Closed	Chr. closed		
PA9	2016–02	2283	893	45.2	6.8	Flye	1	1	Yes	6,389,310	4
PA15	2018–12	2101	960	47.9	6.5		1	1	Yes	6,288,383	4
PA20	2021–12	1793	1328	40.5	3.1		2	0	No	6,830,215	4
PA21	2022–01	2186	1451	42.5	2.8		1	1	Yes	6,508,155	4
PA22	2022–01	2207	834	40.4	7.1		1	1	Yes	6,888,459	4
PA26	2023–08	2356	1376	43.7	3.2		1	1	Yes	6,833,508	4
PA28	2020–08	2225	2408	46.3	4.2		2	2	Yes	6,992,718	4
PA24	2023–01	1644	1302	40.5	3.5	Autocycler	1	1	Yes	6,880,111	4
PA6	2025–04	2286	1347	38.2	4.5	Hybracter	12	0	No	6,959,201	4
PA19	2020–05	2478	1317	41.9	4.0		6	0	No	7,170,394	4
PA23	2022–10	1967	1561	41.9	3.4		12	0	No	7,010,936	4
PA25	2023–03	1906	1375	37.4	4.0		6	0	No	7,100,776	4

* For all isolates, we tried the Flye workflow first. If the contigs were not fully closed by the Flye workflow, we additionally performed Autocycler and Hybracter workflows and used the most contiguous assembly among the outputs of the three workflows. In the Flye workflow, we did long reads-first assembly using Flye v2.9.1 and polished the contigs with short reads using PolyPolish v0.6.0. In the Autocycler workflow, we did long reads-first assembly using Autocycler v0.2.1 and polished the contigs with short reads using PolyPolish v0.6.0. In the Hybracter workflow, we used the ‘hybrid’ mode of Hybracter v.0.11.0.

### Genomic diversity of bacteremic pneumonia isolates

**Fig. 1.**
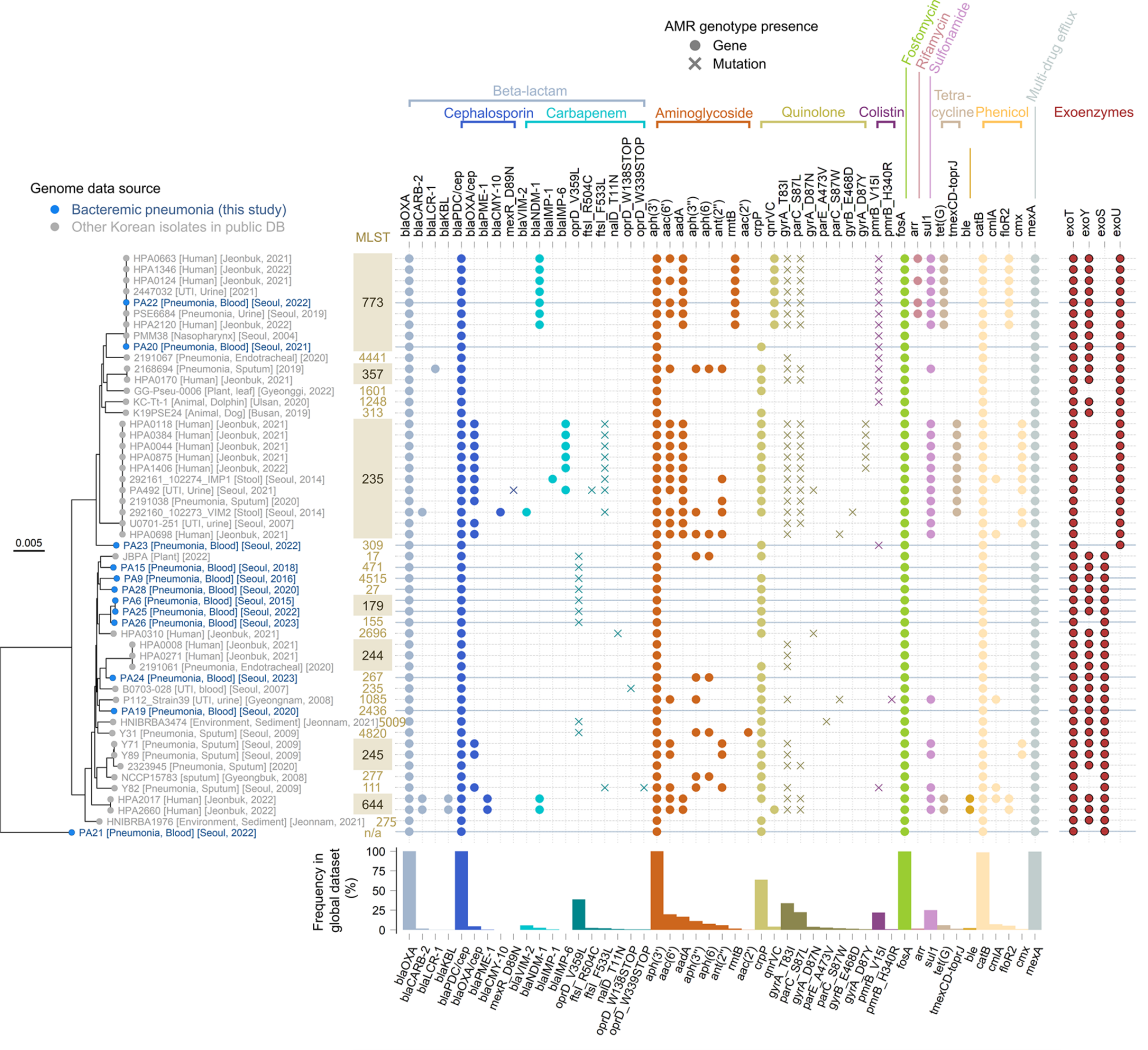
Phylogenetic distribution and AMR genotypes of Korean *P. aeruginosa* isolates. Maximum-likelihood phylogenetic tree on the left side includes the genome sequences of Korean *P. aeruginosa* isolates generated in this study (*n*=12) or retrieved from the NCBI Pathogen Detection database for which the geographic location of isolation was ‘South Korea’ (*n*=41). The tree was reconstructed from concatenated core gene sequences and rooted using *P. paraeruginosa* ATCC 9027 as an outgroup. Isolates from bacteremic pneumonia analysed in this study are indicated in blue, while other Korean isolates from the public database are shown in grey. The presence or absence of AMR genotypes across isolates is shown adjacent to the phylogeny. Filled circles indicate acquired resistance genes and crosses (**x**) indicate point mutations. The bar plot at the bottom represents the frequency of each AMR genotype in a global dataset of *P. aeruginosa* genomes (*n*=46,994). The presence of exoenzyme-encoding genes (i.e. T3SS effectors, known to facilitate systemic infection) in each isolate is shown on the right side. The tree part was plotted using the ggtree package; the two-dimensional plot for genotype presence and the bar plot for global frequency of genotype were generated using the ggplot package.

The 12 isolates from severe bacteremic pneumonia were assigned to nine different STs, with one isolate (PA21) remaining unassigned by the current MLST database (as of 19 October 2025). Only two STs were represented by more than one isolate, namely ST773 (PA20 and PA22) and ST179 (PA6 and PA25), while the remaining isolates belonged to distinct STs. In a core genome phylogenetic tree reconstructed from the 12 bacteremic pneumonia isolates and 41 publicly available *P. aeruginosa* genomes selected from the species-wide public dataset based on the geographic location of ‘South Korea’, the bacteremic pneumonia isolates were distributed across multiple lineages rather than forming a distinct cluster (Fig. 1).

### Resistome and virulence profiles

AMR genotypes varied substantially both among sequence types and within individual sequence types (Fig. 1). Carbapenemase genes were detected in only one isolate, PA22 (ST773), which harboured *bla*_NDM-1_ together with *bla*_OXA-796_ and intrinsic β-lactamases. In the remaining 11 bacteremic pneumonia isolates, carbapenemase genes were not detected. Instead, V359L mutation in OprD, known as a chromosomal carbapenem resistance mechanism, was detected in 6 of 11 carbapenemase-negative isolates. The V359L substitution was notably highly prevalent in the global *P. aeruginosa* genome dataset (38.7%). When we inspected the global genome datasets for the STs containing the V359L mutation-carrying isolates from this study, the V359L mutation was nearly universal in ST27 (661/683, 96.8%), ST155 (796/815, 97.7%), ST179 (1040/1061, 98.0%), but was relatively low in ST471 (4/79, 5.1%). Aminoglycoside resistance determinants other than *aph*(3'), such as *aac*(6'), *aph*(3''), *aadA* and *aph*(6) together with *sul1* were not consistently associated with specific sequence types and were distributed sporadically across the phylogeny. Mutations associated with fluoroquinolone resistance in *gyrA* and *parC* were observed in multiple isolates (Fig. 1).

Analysis of T3SS-associated exotoxin genotypes revealed that three isolates were *exoU*-positive/*exoS*-negative while eight isolates were *exoU*-negative/*exoS*-positive (Fig. 1). The metallo-β-lactamase–producing isolate PA22 exhibited an *exoU*-negative/*exoS*-positive genotype. The core genome-based phylogeny showed clear segregation of isolates according to *exoU* and *exoS* genotypes which is consistent with previous reports.

### Global population structure of ST773

**Fig. 2.**
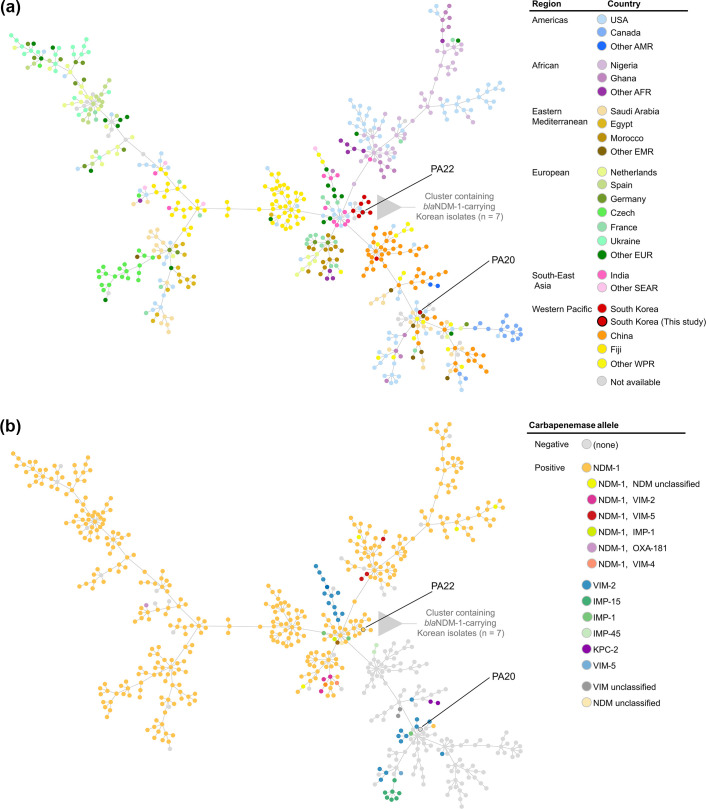
Global population structure and geographic distribution of *P. aeruginosa* ST773 and distribution of carbapenemase alleles. Both panels show the same minimum spanning tree of *P. aeruginosa* ST773 genomes (*n*=598) constructed from cgMLST allele profiles. The cgMLST scheme was defined from the input ST773 genomes using chewBBACA v3.5.3 and the minimum spanning tree was generated using the goeBURST full minimum spanning tree algorithm in PHYLOViZ v2.0 and visualized by Cytoscape v3.10.4. Each node represents a genome and edges indicate allelic differences between profiles. Two bacteremic pneumonia isolates analysed in this study are indicated with text labels. (**a**) Nodes are coloured according to the country of origin illustrating the global geographic distribution of ST773 genomes. (**b**) Nodes are coloured according to the carbapenemase alleles identified in each genome. Carbapenemases were defined as β-lactamase enzymes annotated as conferring resistance to carbapenems based on NCBI AMRFinderPlus v4.0.23 with the database version released on 16 July 2025.

Among the bacteremic pneumonia isolates collected in this study, PA22 (with *bla*_NDM-1_) was the only carbapenemase-positive isolate and belonged to ST773 whereas the other ST773 isolate, PA20, did not carry a metallo-β-lactamase gene. This genotypic distinction was consistent with the antimicrobial susceptibility testing results in which PA22 was the only carbapenem-resistant and multidrug-resistant isolate (Table S2). The coexistence of carbapenemase-positive and carbapenemase-negative ST773 isolates within this cohort led us to investigate whether these isolates were closely related and whether the resistance genes of PA22 reflected recent acquisition within a local ST773 background or placement within a broader predominantly resistant ST773 lineage. Because these questions could not be resolved from the local cohort alone, we constructed a minimum spanning tree based on cgMLST, including all publicly available ST773 genomes from the NCBI Pathogen Detection database (*n*=596) together with PA20 and PA22 (Fig. 2).

Geographically ST773 isolates were widely distributed across multiple regions, including East Asia (South Korea and China), Europe (e.g. the Netherlands, Spain and Germany) and North America (United States and Canada). Of the nine Korean ST773 isolates analysed, seven isolates, including PA22 carried *bla*_NDM-1_ and formed a tight cluster within the NDM-1-positive lineage with cgMLST allele distance of 6–15 from PA22 (Fig. 2). This cluster also contained two isolates from the United States, both collected in 2025, each with an allele distance of 10 from PA22. The two Korean isolates that lacked carbapenemases were distant from this cluster and from each other; PA20 was located separately within the carbapenemase-negative lineage.

Next, we plotted carbapenemase allele profiles on the minimum spanning tree (Fig. 2b). Among the ST773 isolates, *bla*_NDM-1_ was the most prevalent carbapenemase (385/598, 64.4%) followed by *bla*_VIM-2_ (3.9%) and *bla*_IMP-15_ (1.2%). Co-occurrence of *bla*_NDM-1_ with additional carbapenemase genes was also observed in a subset of isolates. The carbapenemase-negative isolates comprised the second most prevalent genotype (26.1%) next to the *bla*_NDM-1_ isolates. We found that the *bla*_NDM-1_–positive isolates and the carbapenemase-negative isolates diverged by a deep branch of the minimum spanning tree, suggesting lineage-level differentiation. Other carbapenemase variants were observed sporadically and tended to form small clusters. In the context of this deep branching divergence within ST773, PA22 was located in a *bla*_NDM-1_-positive lineage whereas PA20 was in a carbapenemase-negative lineage.

### *bla*_OXA_ allele diversity and integron-mediated acquisition in ST773

**Fig. 3.**
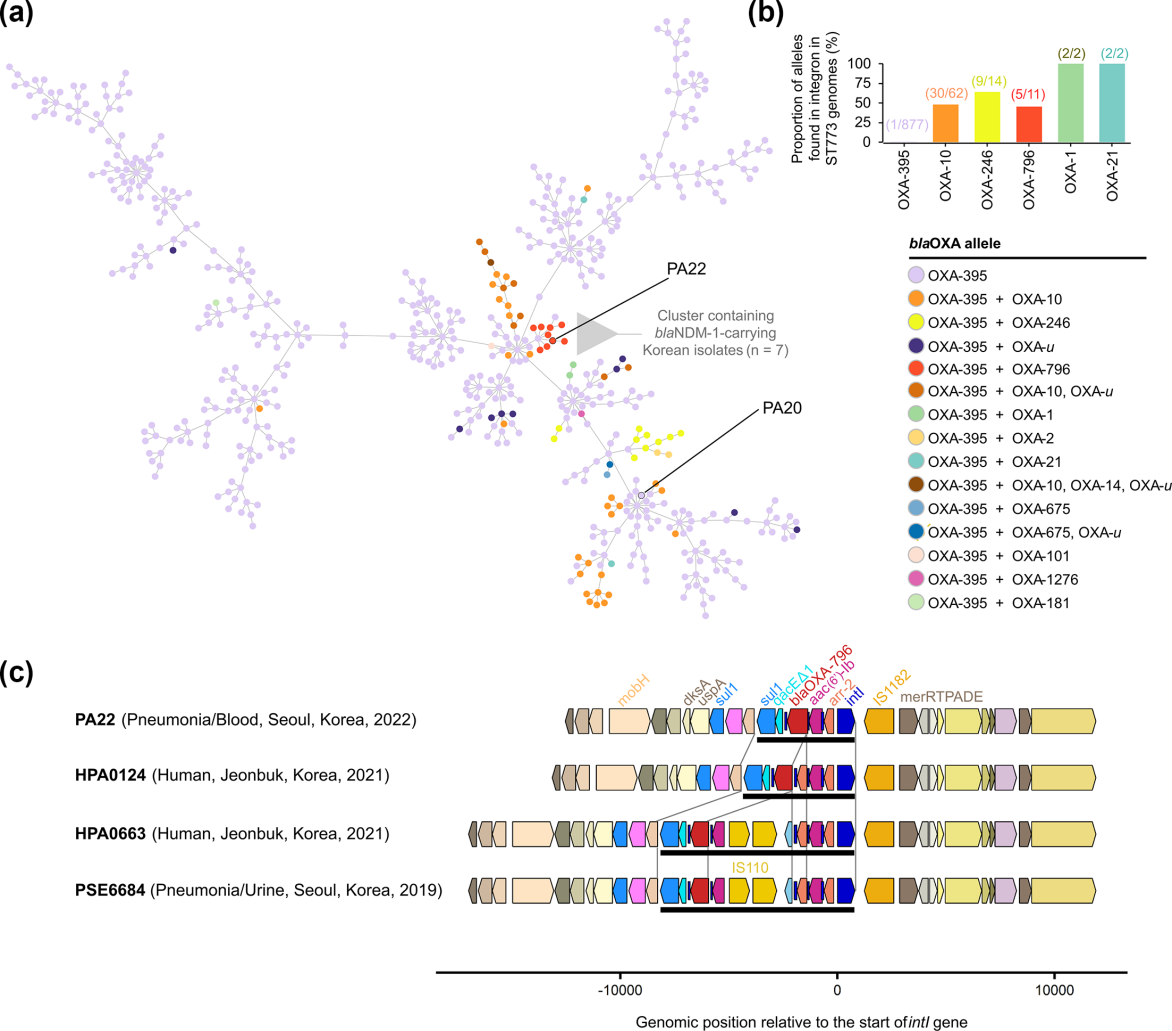
Clonal distribution and integron association of *bla*_OXA_ alleles in *P. aeruginosa* sequence type 773. (**a**) Minimum spanning tree of *P. aeruginosa* ST773 genomes (*n*=598) based on cgMLST allele profiles, as shown in Fig. 2. Nodes are coloured according to the *bla*_OXA_ alleles identified in each genome based on annotation using NCBI AMRFinderPlus v4.0.23 with the database version released on 16 July 2025. (**b**) Proportion of *bla*_OXA_ alleles located within integron structures, as identified using IntegronFinder v2.0.5. Only the alleles detected within integrons in at least one genome are shown. Numbers above bars indicate the number of genomes with integron-associated alleles relative to the total number of genomes carrying the allele. (**c**) The genomic maps of *bla*_OXA-796_-containing integrons and their surrounding regions identified in ST773 genomes, including the PA22 bacteremic pneumonia isolate. Genomic coordinates are displayed relative to the start position of the integrase gene (intI1). The genomic maps were generated using a custom R script.

Another difference in the β-lactamase repertoire of the two ST773 bacteremic pneumonia isolates (PA22 and PA20) was that PA22 harboured a secondary *bla*_OXA_ allele, *bla*_OXA-796_, in addition to the shared *bla*_OXA-395_ allele. To investigate the evolutionary pathway underlying this variation, we examined the distribution of *bla*_OXA_ within ST773 using the cgMLST-based minimum spanning tree annotated with *bla*_OXA_ allele profiles (Fig. 3a). The majority of ST773 isolates carried only the intrinsic *bla*_OXA-395_ allele. However, a subset of isolates harboured additional acquired *bla*_OXA_ variants in combination with *bla*_OXA-395_. These acquired variants included *bla*_OXA-10_, *bla*_OXA-246_, *bla*_OXA-796_, *bla*_OXA-1_, *bla*_OXA-2_ and *bla*_OXA-21_. Notably, isolates sharing identical combinations of *bla*_OXA_ alleles tended to cluster together in the phylogeny, suggesting clonal dissemination of specific resistance configurations. The cluster containing PA22, other *bla*_NDM-1_-positive Korean isolates and two isolates from the United States was characterized by co-carriage of *bla*_OXA-395_ and *bla*_OXA-796_ (Fig. 3a).

On the genomic assembly of PA22, the *bla*_OXA-796_ was located inside a class 1 integron’s gene cassette array. To assess the association between various *bla*_OXA_ alleles and integron structures in ST773, we quantified the proportion of each allele’s occurrence within integrons (note that the association could be falsely negative in short reads-only draft genomes) (Fig. 3b). The intrinsic *bla*_OXA-395_ of ST773 was rarely found within integrons, whereas the other acquired *bla*_OXA_ alleles showed strong association with integrons. In particular, substantial proportions of *bla*_OXA-1_ (two isolates had this gene in integron/two isolates in total carried this gene), *bla*_OXA-21_ (2/2), *bla*_OXA-10_ (30/62), *bla*_OXA-246_ (9/14) and *bla*_OXA-796_ (5/11) were found in class 1 integron.

Next, we examined the genetic context of *bla*_OXA-796_ more closely within the major Korean ST773 cluster (Fig. 3c). In PA22 and the three closely related Korean isolates from public databases (HPA0124, HPA0663 and PSE6684), *bla*_OXA-796_ was located within a class 1 integron gene cassette array downstream of the *intI1* integrase gene. The cassette array included *sul1*, *aac*(6')-Ib and *arr*-2 in addition to *bla*_OXA-796_, comprising a multidrug resistance locus. The integron was flanked by a mercury resistance operon (*merRTPADE*), and the overall genomic organization was highly conserved across the four isolates. However, variation in the insertion sites of mobile elements, including IS1182 and IS110, was observed, indicating structural diversification from a shared ancestral integron.

## Discussion

This study reports the genomic characterization of *P. aeruginosa* isolates from severe bacteremic pneumonia using hybrid long read and short read sequencing, revealing that these life-threatening infections were caused by phylogenetically diverse and mostly carbapenemase-negative lineages. Within this resistance-unselected cohort, an ST773 isolate PA22 was distinguished as the only carbapenem-resistant and multidrug-resistant isolate, thereby motivating a focused analysis of its population-genetic and mobile-element context. The global ST773 analysis showed that PA22 was not closely related to the carbapenemase-negative ST773 isolate PA20, but was positioned within the *bla*_NDM-1_-positive subclone of ST773 and clustered with previously reported *bla*_NDM-1_-positive isolates from Korea, which characteristically carry a *bla*_OXA-796_-containing class 1 integron with evidence of ongoing structural diversification among closely related isolates. These findings exemplify that resistance-unselected surveillance of severe infection can reveal the emergence of high-risk or resistant clones, while long-read assembly enables elucidation of resistome diversification driven by mobile genetic elements within emerging clones.

The 12 bacteremic pneumonia isolates were distributed across nine distinct STs and did not form a single phylogenetic cluster, demonstrating that severe bacteremic pneumonia is not driven by a specific high-risk clone but can arise from diverse genetic backgrounds. Analysis of T3SS-associated exotoxin genotypes revealed a mutually exclusive distribution of *exoU* and *exoS* that was concordant with the core genome phylogeny, consistent with previous observations [10, 47]. The *exoU*-positive genotype has been associated with greater cytotoxicity and poorer clinical outcomes in *P. aeruginosa* infections [48]. Notably, PA22 exhibited an *exoS*-positive/*exoU*-negative genotype yet caused severe bacteremic pneumonia and simultaneously harboured *bla*_NDM-1_. Previous studies have reported that carbapenemase carriage is more frequently observed among *exoU*-positive lineages, such as ST357 [49], ST463 [50] and ST235 [51, 52]. In this context, our report on PA22 represents a case in which high-level carbapenem resistance has been acquired within an *exoS*-positive lineage, suggesting that the convergence of virulence and resistance is not confined to *exoU*-positive clones and that *exoS*-positive lineages acquiring carbapenemases may also pose significant infection control concerns.

Among the *P. aeruginosa* STs that have been reported as major carbapenemase-producing clones in Korea, such as ST235 associated with *bla*_IMP-6_, ST773 associated with *bla*_NDM-1_ and ST644 associated with *bla*_NDM-1_ [16, 53], only ST773 was represented in this study, whereas ST235 and ST644 were not detected. The intrinsic resistance determinants shared across our isolates, including chromosomal *bla*_OXA-395_ and *ampC* β-lactamase, were consistent with those reported in previous studies. However, a notable difference was the identification of the V359L substitution in OprD, an outer membrane porin, as the predominant carbapenem resistance-associated genotype in carbapenemase-negative isolates in our study. Previous Korean studies have primarily reported frameshift mutations in *oprD* [54] or loss of *oprD* by structural variation [55] as the major mechanism of OprD inactivation-mediated resistance. In contrast, the V359L substitution was nearly universal within specific STs in the global dataset – e.g. ST27 (96.8%), ST155 (97.7%), ST179 (98.0%) and ST4515 (100%) – raising the possibility that this substitution represents a fixed ancestral trait of these lineages rather than a *de novo* resistance mutation. Although the mutation OprD V359L has been associated with CRPA, functional significance of V359L remains to be elucidated. The detection of only a single NDM-1-producing isolate in this study likely reflects the inclusion criteria restricted to bacteremic pneumonia isolates regardless of carbapenem susceptibility, rather than the overall prevalence of carbapenemase-producing strains. Indeed, a recent multicentre study reported that NDM-producing ST773 accounted for 42.9% of carbapenemase-producing *P. aeruginosa* isolates in Korea [53], indicating that the frequency of NDM-1-producing strains would be substantially higher in studies specifically targeting carbapenem-resistant isolates.

Within the global ST773 population, the cgMLST-based minimum spanning tree revealed a deep bifurcation separating *bla*_NDM-1_-positive and carbapenemase-negative lineages. Korean isolates were distributed into two distinct clusters: a larger cluster containing PA22 that was phylogenetically proximal to isolates from the United States, and a second cluster containing PA20 that belonged to the carbapenemase-negative lineage. The proximity between the PA22-containing Korean cluster and United States isolates indicates that closely related genomes are present in the public ST773 dataset; however, such interpretation is limited due to outbreak-driven sequencing data, over-representation of some countries and lack of epidemiological metadata. Cross-border spread of NDM-1-producing ST773 has been documented in other contexts, including the clonal dissemination from Ukrainian patients to hospitals in the Netherlands and Spain during 2022 [56]. The rapid epidemiological trajectory of NDM-1-producing ST773 in Korea, reflected in a 2023 report describing ST773 as a recently emerging clone [16] and a 2025 report ranking it as the top prevalent CRPA strain [53], underscores the urgency of monitoring this lineage regardless of geographic region. A single-centre study from a similar period reported that 88.1% of CRPA isolates belonged to either ST773 (50.8%) or ST235 (37.3%), harbouring *bla*_NDM-1_ and *bla*_IMP-6_, respectively [57]. A multicentre study published in 2025 further confirmed this trend, with carbapenemase genes detected in 73.0% of CRPA isolates; *bla*_NDM_ ST773 accounted for 42.9%, *bla*_NDM_ ST644 for 14.3% and *bla*_IMP_ ST235 for 11.1% [53].

Carriage of *bla*_OXA-796_ in addition to the intrinsic *bla*_OXA-395_ was a feature that distinguishes the isolate PA22 from other NDM-1-producing ST773 isolates that typically carry *bla*_OXA-395_ alone. Across the ST773 population, acquired *bla*_OXA_ alleles were predominantly associated with integron-mediated acquisition, in contrast to the intrinsic *bla*_OXA-395_, which was rarely integron-associated. In PA22 and three closely related Korean isolates from public databases (HPA0124, HPA0663 and PSE6684), the *bla*_OXA-796_ gene was embedded within a class 1 integron gene cassette array containing *sul1*, *aac*(6')-Ib and *arr*-2, flanked by a mercury resistance operon (*merRTPADE*). This overall genomic organization was highly conserved across the four isolates; however, insertion of IS110 inside the gene cassette array was observed only in two isolates (HPA0663 and PSE6684), indicating ongoing structural diversification since the acquisition of integron in a recent past. Some closely related isolates harboured additional resistance determinants within the integron, suggesting that the cassette array is undergoing continuous diversification through gene cassette acquisition and deletion [58].

This study has several limitations. First, 5 of the 12 isolates did not yield fully circularized genome assemblies, resulting in 2–12 unclosed contigs. Given that the correct number (i.e. four copies) of the ribosomal RNA-encoding operons have been resolved into separate locations in the assembly [45, 46], the failures in those five isolates may be attributable to repetitive sequences, such as ICEs or composite transposons that are longer than rRNA operons (ca. 4.5 kbp). Consequently, the completeness of plasmid and mobile genetic element analyses may have been affected in these isolates. Second, this was a single-centre study with a limited number of isolates (*n*=12), which may not fully represent the epidemiology of *P. aeruginosa* bacteremic pneumonia across Korea. Multi-centre prospective studies are needed to comprehensively characterize the population structure and transmission dynamics of this pathogen in invasive infections.

In conclusion, this study demonstrates that severe bacteremic pneumonia caused by *P. aeruginosa* is driven by phylogenetically diverse lineages with heterogeneous virulence and resistance profiles, highlighting the need for comprehensive, unbiased genomic surveillance. Additionally, the ongoing resistance evolution of the rapidly emerging NDM-1-producing ST773 clone underscores the growing therapeutic challenge this lineage may present in the future. Multi-centre studies are, therefore, warranted to fully elucidate the population structure and transmission dynamics of *P. aeruginosa* strains causing invasive pneumonia.

## Supplementary material

10.1099/mgen.0.001815 Supplementary Material 1.

10.1099/mgen.0.001815 Supplementary Material 2.

## Abbreviations

AMR: antimicrobial resistance
cgMLST: core genome multilocus sequence typing
CRPA: carbapenem-resistant P. aeruginosa
HAP: hospital-acquired pneumonia
ICE: integrative and conjugative element
ICU: intensive care unit
IS: insertion sequence
MLST: multilocus sequence typing
ST: sequence type
T3SS: type III secretion system
VAP: ventilator-associated pneumonia
WGS: whole-genome sequencing

## References

[1] Guillamet CV, Vazquez R, Noe J, Micek ST, Kollef MH (2016). A cohort study of bacteremic pneumonia: the importance of antibiotic resistance and appropriate initial therapy?. Medicine.

[2] Hoban DJ, Biedenbach DJ, Mutnick AH, Jones RN (2003). Pathogen of occurrence and susceptibility patterns associated with pneumonia in hospitalized patients in North America: results of the SENTRY Antimicrobial Surveillance Study (2000). Diagn Microbiol Infect Dis.

[3] Quartin AA, Scerpella EG, Puttagunta S, Kett DH (2013). A comparison of microbiology and demographics among patients with healthcare-associated, hospital-acquired, and ventilator-associated pneumonia: a retrospective analysis of 1184 patients from a large, international study. BMC Infect Dis.

[4] Chang Y, Jeon K, Lee S-M, Cho Y-J, Kim YS (2021). The distribution of multidrug-resistant microorganisms and treatment status of hospital-acquired pneumonia/ventilator-associated pneumonia in adult intensive care units: a prospective cohort observational study. J Korean Med Sci.

[5] Curran CS, Bolig T, Torabi-Parizi P (2018). Mechanisms and targeted therapies for *Pseudomonas aeruginosa* lung infection. Am J Respir Crit Care Med.

[6] Park S-Y, Park HJ, Moon SM, Park K-H, Chong YP (2012). Impact of adequate empirical combination therapy on mortality from bacteremic *Pseudomonas aeruginosa* pneumonia. BMC Infect Dis.

[7] Albasanz-Puig A, Durà-Miralles X, Laporte-Amargós J, Mussetti A, Ruiz-Camps I (2022). Effect of combination antibiotic empirical therapy on mortality in neutropenic cancer patients with *Pseudomonas aeruginosa* pneumonia. Microorganisms.

[8] Le Berre R, Nguyen S, Nowak E, Kipnis E, Pierre M (2011). Relative contribution of three main virulence factors in *Pseudomonas aeruginosa* pneumonia. Crit Care Med.

[9] Peña C, Cabot G, Gómez-Zorrilla S, Zamorano L, Ocampo-Sosa A (2015). Influence of virulence genotype and resistance profile in the mortality of *Pseudomonas aeruginosa* bloodstream infections. Clin Infect Dis.

[10] Ozer EA, Nnah E, Didelot X, Whitaker RJ, Hauser AR (2019). The population structure of *Pseudomonas aeruginosa* is characterized by genetic isolation of *exoU*+ and *exoS*+ lineages. Genome Biol Evol.

[11] Shaver CM, Hauser AR (2004). Relative contributions of *Pseudomonas aeruginosa* ExoU, ExoS, and ExoT to virulence in the lung. Infect Immun.

[12] Berube BJ, Rangel SM, Hauser AR (2016). *Pseudomonas aeruginosa*: breaking down barriers. Curr Genet.

[13] Valik JK, Giske CG, Hasan B, Gozalo-Margüello M, Martínez-Martínez L (2024). Genomic virulence markers are associated with severe outcomes in patients with *Pseudomonas aeruginosa* bloodstream infection. Commun Med.

[14] Oliver A, Rojo-Molinero E, Arca-Suarez J, Beşli Y, Bogaerts P (2024). *Pseudomonasaeruginosa* antimicrobial susceptibility profiles, resistance mechanisms and international clonal lineages: update from ESGARS-ESCMID/ISARPAE group. Clin Microbiol Infect.

[15] Yoo JS, Yang JW, Kim HM, Byeon J, Kim HS (2012). Dissemination of genetically related IMP-6-producing multidrug-resistant *Pseudomonas aeruginosa* ST235 in South Korea. Int J Antimicrob Agents.

[16] Choi YJ, Kim YA, Junglim K, Jeong SH, Shin JH (2023). Emergence of NDM-1-producing *Pseudomonas aeruginosa* sequence type 773 clone: shift of carbapenemase molecular epidemiology and spread of 16S rRNA methylase genes in Korea. Ann Lab Med.

[17] Kim T, Hong Y-K, Park S, Jung J, Cho O-H (2025). Genetic epidemiology and antimicrobial susceptibilities of carbapenem-resistant *Pseudomonas aeruginosa* isolates from a multicenter study in Korea. Infect Chemother.

[18] Peña C, Cabot G, Gómez-Zorrilla S, Zamorano L, Ocampo-Sosa A (2015). Influence of virulence genotype and resistance profile in the mortality of *Pseudomonas aeruginosa* bloodstream infections. Clin Infect Dis.

[19] Mandell LA, Wunderink RG, Anzueto A, Bartlett JG, Campbell GD (2007). Infectious Diseases Society of America/American Thoracic Society consensus guidelines on the management of community-acquired pneumonia in adults. Clin Infect Dis.

[20] Chen S (2025). fastp 1.0: an ultra-fast all-round tool for FASTQ data quality control and preprocessing. *Imeta*.

[21] Steinig E, Coin L (2022). Nanoq: ultra-fast quality control for nanopore reads. J Open Source Softw.

[22] Kolmogorov M, Yuan J, Lin Y, Pevzner PA (2019). Assembly of long, error-prone reads using repeat graphs. Nat Biotechnol.

[23] Wick RR, Holt KE (2022). Polypolish: short-read polishing of long-read bacterial genome assemblies. PLoS Comput Biol.

[24] Bouras G, Grigson SR, Papudeshi B, Mallawaarachchi V, Roach MJ (2024). Dnaapler: a tool to reorient circular microbial genomes. J Open Source Softw.

[25] Wick RR, Howden BP, Stinear TP (2025). Autocycler: long-read consensus assembly for bacterial genomes. Bioinformatics.

[26] Bouras G, Houtak G, Wick RR, Mallawaarachchi V, Roach MJ (2024). Hybracter: enabling scalable, automated, complete and accurate bacterial genome assemblies. Microb Genom.

[27] Astashyn A, Tvedte ES, Sweeney D, Sapojnikov V, Bouk N (2024). Rapid and sensitive detection of genome contamination at scale with FCS-GX. Genome Biol.

[28] Silva M, Machado MP, Silva DN, Rossi M, Moran-Gilad J (2018). chewBBACA: a complete suite for gene-by-gene schema creation and strain identification. Microb Genom.

[29] Nascimento M, Sousa A, Ramirez M, Francisco AP, Carriço JA (2017). PHYLOViZ 2.0: providing scalable data integration and visualization for multiple phylogenetic inference methods. Bioinformatics.

[30] Shannon P, Markiel A, Ozier O, Baliga NS, Wang JT (2003). Cytoscape: a software environment for integrated models of biomolecular interaction networks. Genome Res.

[31] Tönnies H, Prior K, Harmsen D, Mellmann A (2021). Establishment and evaluation of a core genome multilocus sequence typing scheme for whole-genome sequence-based typing of *Pseudomonas aeruginosa*. J Clin Microbiol.

[32] Katoh K, Standley DM (2013). MAFFT multiple sequence alignment software version 7: improvements in performance and usability. Mol Biol Evol.

[33] Minh BQ, Schmidt HA, Chernomor O, Schrempf D, Woodhams MD (2020). IQ-TREE 2: new models and efficient methods for phylogenetic inference in the genomic era. Mol Biol Evol.

[34] Seemann T (2014). Prokka: rapid prokaryotic genome annotation. Bioinformatics.

[35] Feldgarden M, Brover V, Gonzalez-Escalona N, Frye JG, Haendiges J (2021). AMRFinderPlus and the reference gene catalog facilitate examination of the genomic links among antimicrobial resistance, stress response, and virulence. Sci Rep.

[36] Liu B, Zheng D, Zhou S, Chen L, Yang J (2022). VFDB 2022: a general classification scheme for bacterial virulence factors. Nucleic Acids Res.

[37] Buchfink B, Reuter K, Drost H-G (2021). Sensitive protein alignments at tree-of-life scale using DIAMOND. Nat Methods.

[38] Cantalapiedra CP, Hernández-Plaza A, Letunic I, Bork P, Huerta-Cepas J (2021). eggNOG-mapper v2: functional annotation, orthology assignments, and domain prediction at the metagenomic scale. Mol Biol Evol.

[39] Hernández-Plaza A, Szklarczyk D, Botas J, Cantalapiedra CP, Giner-Lamia J (2023). eggNOG 6.0: enabling comparative genomics across 12 535 organisms. Nucleic Acids Res.

[40] Robertson J, Nash JHE (2018). MOB-suite: software tools for clustering, reconstruction and typing of plasmids from draft assemblies. Microb Genom.

[41] Néron B, Littner E, Haudiquet M, Perrin A, Cury J (2022). IntegronFinder 2.0: identification and analysis of integrons across bacteria, with a focus on antibiotic resistance in *Klebsiella*. Microorganisms.

[42] Xie Z, Tang H (2017). ISEScan: automated identification of insertion sequence elements in prokaryotic genomes. Bioinformatics.

[43] Liu M, Li X, Xie Y, Bi D, Sun J (2019). ICEberg 2.0: an updated database of bacterial integrative and conjugative elements. Nucleic Acids Res.

[44] Ross K, Varani AM, Snesrud E, Huang H, Alvarenga DO (2021). TnCentral: a prokaryotic transposable element database and web portal for transposon analysis. mBio.

[45] Hartmann RK, Toschka HY, Ulbrich N, Erdmann VA (1986). Genomic organization of rDNA in *Pseudomonas aeruginosa*. FEBS Lett.

[46] Koren S, Phillippy AM (2015). One chromosome, one contig: complete microbial genomes from long-read sequencing and assembly. Curr Opin Microbiol.

[47] Akter T, Stapleton F, Willcox M (2025). Differences in antimicrobial resistance between exoU and exoS isolates of *Pseudomonas aeruginosa*. Eur J Clin Microbiol Infect Dis.

[48] Luo R-G, Miao X-Y, Luo L-L, Mao B, Yu F-Y (2019). Presence of *pldA* and *exoU* in mucoid *Pseudomonas aeruginosa* is associated with high risk of exacerbations in non-cystic fibrosis bronchiectasis patients. Clin Microbiol Infect.

[49] Papagiannitsis CC, Medvecky M, Chudejova K, Skalova A, Rotova V (2017). Molecular Characterization of carbapenemase-producing *Pseudomonas aeruginosa* of Czech origin and evidence for clonal spread of extensively resistant sequence type 357 expressing IMP-7 metallo-β-lactamase. Antimicrob Agents Chemother.

[50] Zhu Y, Kang Y, Zhang H, Yu W, Zhang G (2023). Emergence of ST463 *exoU* -positive, imipenem-nonsusceptible *Pseudomonas aeruginosa* isolates in China. Microbiol Spectr.

[51] Fischer S, Dethlefsen S, Klockgether J, Tümmler B (2020). Phenotypic and genomic comparison of the two most common ExoU-positive *Pseudomonas aeruginosa* clones, PA14 and ST235. mSystems.

[52] Teo JQ-M, Tang CY, Lim JC, Lee SJ-Y, Tan SH (2021). Genomic characterization of carbapenem-non-susceptible *Pseudomonas aeruginosa* in Singapore. Emerg Microbes Infect.

[53] Kim T, Hong Y-K, Park S, Jung J, Cho O-H (2025). Genetic epidemiology and antimicrobial susceptibilities of carbapenem-resistant *Pseudomonas aeruginosa* isolates from a multicenter study in Korea. Infect Chemother.

[54] Kim CH, Kang HY, Kim BR, Jeon H, Lee YC (2016). Mutational inactivation of OprD in carbapenem-resistant *Pseudomonas aeruginosa* isolates from Korean hospitals. J Microbiol.

[55] Sung JY, Cho HH, Kwon KC, Koo SH (2011). Chromosomal mutations in *oprD*, *gyrA*, and parc in carbapenem resistant *Pseudomonas aeruginosa*. Ann Clin Microbiol.

[56] Hernández-García M, González de Aledo M, Ponce-Alonso M, González-Blanco B, Viedma E (2024). Simultaneous clonal spread of NDM-1-producing *Pseudomonas aeruginosa* ST773 from Ukrainian patients in the Netherlands and Spain. *IJID Reg*.

[57] Park Y, Koo SH (2022). Epidemiology, molecular characteristics, and virulence factors of carbapenem-resistant *Pseudomonas aeruginosa* isolated from patients with urinary tract infections. IDR.

[58] Souque C, Escudero JA, MacLean RC (2021). Integron activity accelerates the evolution of antibiotic resistance. Elife.

