# Supplementary material for: Genomic diversity of Pseudomonas aeruginosa causing bacteremic pneumonia in an intensive care unit, with emergence of an OXA-796-producing NDM-1 ST773 isolate
Source: Microb Genom. 2026 Aug 6;12(8):001815. doi: 10.1099/mgen.0.001815 (PMC13446753; doi:10.1099/mgen.0.001815)
Supplement: Supplementary file 1 [file mgen-12-01815-s001.pdf]

Supplementary Table 2. Phenotypic antimicrobial susceptibility profiles of 12 *P. aeruginosa* isolates collected in this study.

Isolate	Antimicrobial susceptibility result									
	**AMK**	**ATM**	**CAZ**	**CIP[a]**	**FEP**	**GEN[b]**	**IPM**	**LVX[a]**	**MEM**	**TZP**
PA9	S	S	S	S	S	S	S	S	S	S
PA15	S	S	S	S	S	S	S	S	S	S
PA20	S	S	S	S	S	S	S	S	S	S
PA21	S	S	S	S	S	S	S	S	S	S
PA22	R	S	R	R	R	R	R	R	R	R
PA26	S	S	S	S	S	S	S	S	S	S
PA28	S	S	S	S	S	S	S	S	S	S
PA24	S	S	S	S	S	S	S	S	S	S
PA6	S	I	S	S	S	S	S	S	S	S
PA19	S	S	S	S	S	S	S	S	S	S
PA23	S	S	S	S	S	S	S	S	S	S
PA25	S	S	S	S	S	S	S	S	S	S

Abbreviations: AMR, antimicrobial resistance; AMK, amikacin; ATM, aztreonam; CAZ, ceftazidime; CIP, Ciprofloxacin; FEP, cefepime; GEN, gentamicin; IPM, imipenem; LVX, levofloxacin; MEM, Meropenem; TZP, piperacillin-tazobactam

[a] Interpreted according to CLSI 2012 criteria.

[b] Interpreted according to EUCAST 2025 criteria.

All other antimicrobial susceptibility interpretations were based on CLSI M100, 35th edition (2025).
